# Exploring the Mechanisms of Differentiation, Dedifferentiation, Reprogramming and Transdifferentiation

**DOI:** 10.1371/journal.pone.0105216

**Published:** 2014-08-18

**Authors:** Li Xu, Kun Zhang, Jin Wang

**Affiliations:** 1 State Key Laboratory of Electroanalytical Chemistry, Changchun Institute of Applied Chemistry, Chinese Academy of Sciences, Changchun, Jilin, China; 2 Department of Chemistry & Physics, State University of New York at Stony Brook, Stony Brook, New York, United States of America; Institute of Medical Biology, Singapore

## Abstract

We explored the underlying mechanisms of differentiation, dedifferentiation, reprogramming and transdifferentiation (cell type switchings) from landscape and flux perspectives. Lineage reprogramming is a new regenerative method to convert a matured cell into another cell including direct transdifferentiation without undergoing a pluripotent cell state and indirect transdifferentiation with an initial dedifferentiation-reversion (reprogramming) to a pluripotent cell state. Each cell type is quantified by a distinct valley on the potential landscape with higher probability. We investigated three driving forces for cell fate decision making: stochastic fluctuations, gene regulation and induction, which can lead to cell type switchings. We showed that under the driving forces the direct transdifferentiation process proceeds from a differentiated cell valley to another differentiated cell valley through either a distinct stable intermediate state or a certain series of unstable indeterminate states. The dedifferentiation process proceeds through a pluripotent cell state. Barrier height and the corresponding escape time from the valley on the landscape can be used to quantify the stability and efficiency of cell type switchings. We also uncovered the mechanisms of the underlying processes by quantifying the dominant biological paths of cell type switchings on the potential landscape. The dynamics of cell type switchings are determined by both landscape gradient and flux. The flux can lead to the deviations of the dominant biological paths for cell type switchings from the naively expected landscape gradient path. As a result, the corresponding dominant paths of cell type switchings are irreversible. We also classified the mechanisms of cell fate development from our landscape theory: super-critical pitchfork bifurcation, sub-critical pitchfork bifurcation, sub-critical pitchfork with two saddle-node bifurcation, and saddle-node bifurcation. Our model showed good agreements with the experiments. It provides a general framework to explore the mechanisms of differentiation, dedifferentiation, reprogramming and transdifferentiation.

## Introduction

A pluripotent undifferentiated cell can differentiate into types of differentiated cells. Each cell type has a specific regulated gene expression. Cellular differentiation is determined by the underlying gene regulatory network during the process of development, which leads the primary cell into its ultimate fate-a particular phenotype. Induced pluripotent stem (iPS) cells provide the opportunity to obtain pluripotent stem cells which potentially have therapeutic uses [1], [2]. Recently many studies have been reported that one type of cells can be converted to another type of functional cells directly [3]–[7]. This is a big step forward in the cell biology since there is no need to create iPS cells first for cell type switching, skipping many intermediate steps. This direct reprogramming technology is called the lineage reprogramming. Thus an adult cell can be reprogrammed directly to new cells as lineage switching. The lineage switching through direct transdifferentiation without going through the iPS state might be applied to regenerative medicine with less risk of cancer. However, it is still challenging to quantify the mechanisms of the differentiation, dedifferentiation, reprogramming and transdifferentiation [3]–[11].

The concept of “epigenetic landscape” was first introduced by Waddington in 1940s [12] The quantifications of the Waddington potential landscape for the process of cell differentiation have been explored recently [13]–[17]. Different valleys represent different cell phenotypes (cell fates) on the cell development potential landscape [13]–[17]. Waddington visualized the undifferentiated state as the local maximum and differentiated states as the local minimum on the landscape [12]. In our landscape picture, the undifferentiated state and differentiated state are both local minima in certain regions of the landscape. Undifferentiated state has relatively low expressions of differentiation mark genes while differentiated state has at least one high expressions of differentiation mark genes. In addition, Waddington believed the differentiation is a downhill process driven by the funneled landscape gradient. In our picture, the differentiation can occur with several different mechanisms, through funneled landscape, through stochastic fluctuations and the probability fluxes even when the landscape is not funneled towards the differentiated states, and through induction.

**Figure 1:**
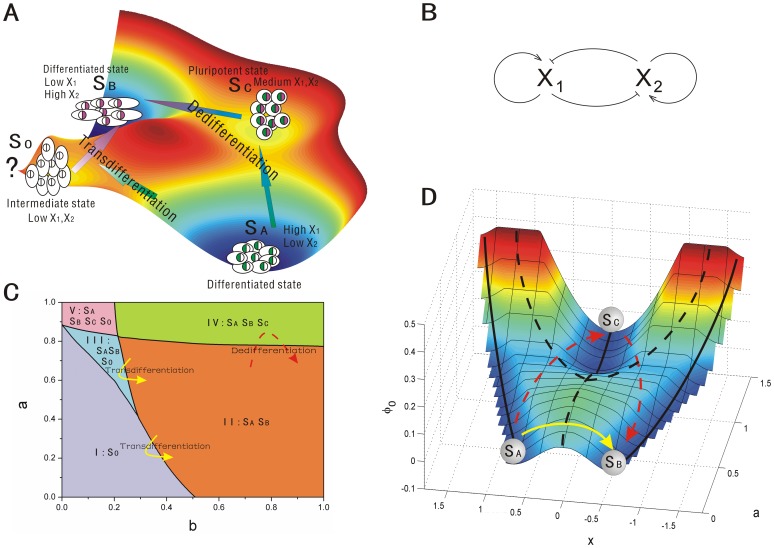
The scheme, phase diagram and intrinsic potential landscape of cell type switchings. A: The scheme of dedifferentiation (including reprogramming and differentiation) and transdifferentiation. B: A model for the gene circuit for cell development. C: The phase diagram for the gene circuit with 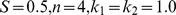. D: The cell fate landscape 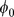 obtained from the Hamilton-Jacobi equation versus 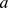 and 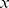, and the phase diagram was drawn on the intrinsic potential landscape with stable states represented by black solid lines and unstable states represented by black dash line. The red dash lines represent the dedifferentiation(reprogramming) and redifferentiation process while the yellow solid lines represents the transdifferentiation process. (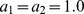, 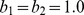, 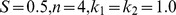.)

For development and differentiation system, we represent a cell as a chemical system having given genomic makeup, with each and every possible phenotype as a potential “state” [18], [19]. This is very much analogous to the notion of a polypeptide, as a chemical molecule, can have many different possible “conformational states”, although each individual protein molecule has only a particular state at a given moment in time. This chemical definition of “the system” is important. Imagine that proteins are defined only through biological functions; then different conformations of a polypeptide will be considered as “different molecules.” Then the notion of spontaneous conformational change would not make sense. Indeed, there are still cell biologists who think different cells from the same person as different cells; rather than as a “same chemical system in different states” [18], [19]. The process of the cell development can be viewed as the system moving from one valley (primary or stem cell phenotype) through bifurcation to another valley (differentiated cell phenotype) on the potential landscape. And the transdifferentiation process can be viewed as the system escaping from one stable differentiated valley to another differentiated valley through certain paths on the potential landscape shown in Figure 1(A). The differentiated cells (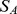) can switch to another lineage cell type (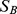) through an explicit pluripotent stable state (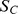). Indirect transdifferentiation mechanism which requires an initial dedifferentiation step 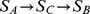 shown in Figure 1(A). It illustrates a differentiated cell (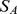) reprogrammed back to a pluripotent state (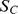) with less differentiated, and then can be re-differentiated to another type of differentiated cell (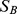) [3], [5], [6]. This is a possible strategy of pluripotent lineage reprogramming while the enhancement of efficiency is required. The underlying process is a transdifferentiation involving a stepwise dedifferentiation. In addition to indirect transdifferentiation, there is another lineage reprogramming approach: the direct transdifferentiation mechanism as 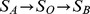 shown in Figure 1(A). Direct transdifferentiation is a mechanism of converting one type of differentiated cells to another type of differentiated cells without undergoing through a pluripotent state or progenitor cell type. The differentiated cells (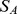) down regulate their own cell-specific genes (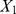) and activate the target cell-specific genes (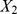), thus they can switch to another lineage cell type (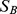) through an explicit intermediate stable state (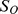) or a series of indeterminate states [3]–[5], [8], [9]. In our study, the intermediate state is defined as an intermediate stable state with low or medium pluripotency and having very low expressions of the differentiation mark genes, while a series of indeterminate states are defined as a series of unstable states with low or medium pluripotency and very low expressions of differentiation mark genes in the course of lineage switching. Sridharan et al [20] showed that partially reprogrammed cells as an intermediate stage of the reprogramming process can switch to the completely reprogrammed iPS state. Thus the states of partially reprogrammed cells may exist along the paths from a differentiated state 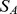 or 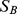 to iPS state 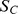. The research by Mikkelsen [21] showed that partially reprogrammed cells can be trapped at a common intermediate state. Thus the states of partially reprogrammed cells may exist along the paths from a differentiated state 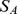 to another differentiated state 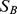 through an intermediate 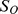 or indeterminate states. These intermediate state and indeterminate states may have certain expressions of stem cell marker genes and thus can be viewed as partially reprogrammed cells. This is supported by the observation that fibroblast cells specific genes are efficiently silenced and the embryonic reprogramming is not fully induced in partially reprogrammed cells [20]. We believe that different experimental and environmental conditions can lead to quite different results and change the topological structure of the potential landscape [20], [21]. The partially reprogrammed cells may be trapped in certain regions in the gene expression space.

In this study, we term direct transdifferentiation as transdifferentiation and indirect transdifferentiation requiring an initial dedifferentiation or reprogramming step as dedifferentiation. The goal of regenerative medicine can potentially be realized through the processes of differentiation, dedifferentiation, reprogramming and transdifferentiation [4]. Here we use cell type switchings short for the terms “differentiation, dedifferentiation, reprogramming, and transdifferentiation”. Recent advances have shown that there are three possible driving forces for cell type switchings: (1) *Stochastic Fluctuations*. Cells choose their pathways of differentiation stochastically in the process of development without apparent regards to environment or history [22]. Some studies in cell development reveal that intrinsic stochasticity is an important mechanism for development [22]. The extrinsic fluctuations are also expected to play a role in cell development. Thus the fluctuations can be a driving force for the processes of cell type switchings. (2) *Gene Regulation*. cell type switchings can be achieved by the change of regulation strengths of their lineage specific genes in many studies [6], [8], [9], [14], [15]. (3) *Induction*. Lineage specific cells can be reprogrammed to a pluripotent state through over-expressions of some defined transcription factors [23], [24]. Transfection of certain cell specific genes into the primary cells, and over-expressions of the target lineage specific genes as well as certain stem cell-associated genes can induce the processes of cell type switchings.

**Figure 2:**
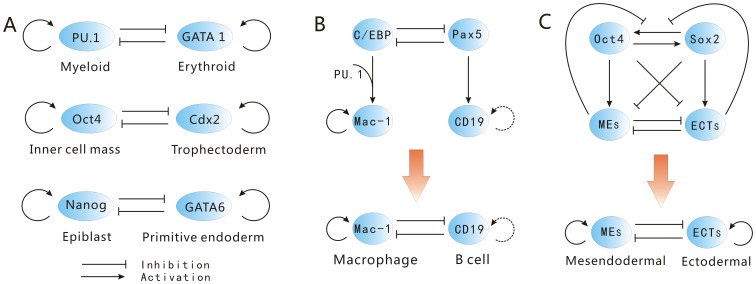
The gene circuits of mutual antagonism and self activation. A: The interaction of 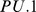 and 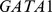 in determining myeloid cell or erythroid cell, 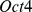 and 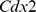 in determining inner cell mass or trophectoderm, 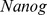 and 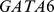 in determining epiblast or primitive endoderm. B: Scheme for the gene circuit of B cell to macrophage conversion. The dashed lines indicate uncertainty. C: Scheme for the gene circuit in determining mesendodermal and ectodermal.

Given the three driving forces for cell fate decision making, it is still challenging on how to quantify the processes of cell type switchings on the landscape, and how to connect them to experiments. These processes of cell type switchings are controlled by their underlying gene regulatory network. The lineage-specific transcription factors play a critical role in the processes of cell type switchings. In this study, we explored a simple cell differentiation network module with autoregulation and mutual antagonism between transcription factors (lineage-specific genes) [15], [17], which exists in many cell differentiation processes, shown in Figure 1(B). The lineage-specific genes can strongly instruct the cellular lineage choice. The circuit is composed of a pair of self activating autoregulation and mutual inhibiting cross-antagonism cell-specific genes 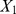 and 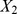 [15], [17]. In iPSC or ESC (embryonic stem cell), pluripotent genes are often highly expressed, and most lineage related genes are off. However, there are examples of gene regulatory circuits with the same architecture in our study which control binary decisions at branch points of cell differentiation in multi-potent cells. Such mutual antagonism gene circuit modules (where the self activation can also be indirect) in binary branch points of cell lineage commitment can often be found. A lot of studies have explored the primed multipotent common myeloid progenitor (CMP) can differentiate to either myeloid cell or erythroid cell in blood cell formation by mutual antagonism interaction of transcription factor gene 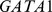 and 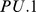 shown in Figure 2(A) [25], [26]. 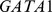 and 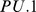 are both self-activated. In the genetic regulation of the inner cell mass/trophectoderm lineage decision, 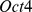 represses expression of 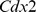, and 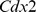 represses expression of 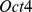 to allow the segregation of inner cell mass and trophectoderm lineages [27], [28]. 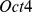 and 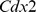 are mutual inhibited and self-activated [27], [28] shown in Figure 2(A). In the genetic regulation of the epiblast/primitive endoderm lineage decision, antagonism between Nanog and Gata6 results in segregation of primitive endoderm and epiblast within the inner cell mass [27], [29], [30] shown in Figure 2(A). 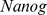 and 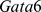 are also both self-activated [30]. These three circuits all can be viewed as 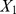 and 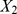 in our network.

We will study this key network module to uncover the underlying functional mechanisms of cell type switchings. The phase diagram in Figure 1(C) suggests that the system can have five different phase regions, each of which has different underlying landscapes with different distribution of valleys. Furthermore, we show how stochastic fluctuation, gene regulation and induction induce the cell type switchings. The potential landscape and flux both direct the processes of cell type switchings. Probability flux provide a curling force breaking the detailed balance and lead the biological paths of cell type switchings to be deviated from the paths obtained by steepest descent gradient of the landscape. The forward and backward paths of cell type switchings are irreversible, without passing through the saddle point. Furthermore, the flux can become the main driving force for cell type switching when the landscape is not biased towards the specific processes [16], [31]. Barrier height and dynamic transition speed are used to quantify the global stability of the landscape topography. The stability here represents the ability for a cell to stay at a certain cell type state against certain fluctuations. In practice, the fluctuations in some cases maybe small but never zero. We uncover and classify four mechanisms of cell type switchings: super-critical pitchfork bifurcation, sub-critical pitchfork bifurcation, sub-critical pitchfork with two saddle-node bifurcation, and saddle-node bifurcation.

## Results and Discussions

### I. The model of cell fate network

We start with gene circuit module for typical differentiation. The gene regulatory circuit for cell fate decision has two mutual repression and self-activation lineage-specific transcription factors: 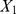 and 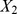 shown in Figure 1(B). It is more complete to consider three or more gene system. But the challenge is that a network with more genes requires more parameters to describe and therefore much bigger search space to explore exhaustively for uncovering the underlying mechanisms. Furthermore, with more genes, it is more difficult to visualize the results. The two gene system we considered is the simplest to exhaustively and effectively explore the underlying mechanism in parameter space [15]–[17], [25]. We would like to use this model to explore the basic underlying mechanisms. The dynamics of this circuit is described by a set of two-variable ordinary differential equations below, with the rate of expression change for these two genes:
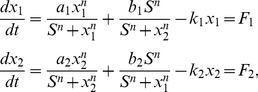
(1)where 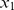 and 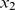 are the time-dependent expressions of the two cell-specific transcription factors 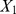 and 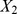 [15], [17], [25]. Parameter 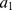 and 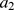 are the self activation strength of the transcription factors 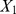 and 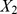 respectively. 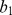 and 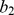 are the strength of the mutual repression for transcription factors 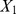 and 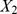 respectively. 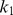 and 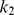 are the first-order degradation rate for 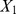 and 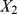 respectively [15], [17], [25]. 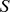 represents the threshold (inflection point) of the sigmoidal functions, i.e., the minimum concentration needed for appreciable changes, and 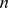 is the Hill coefficient which represents the cooperativity of the regulatory binding and determines the steepness of the sigmoidal function. For simplicity, we do not include studies of all the different parameters of 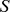 and 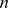 in the main text. We included the studies in the supporting information. We show the phase diagrams for varying these parameters in Figure S1 in File S1. We can see varying these parameters can also lead to bi-stable states or tri-stable states and also the phase transitions. In the main text, the parameters for Hill function and degradation rate for 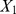 and 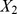 are specified as: 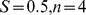, and 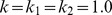 [15]–[17], [25]. In this section, we assume the symmetric situation 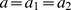 and 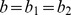. Although the values of parameters can be different in organisms under different circumstance, the mathematical model here describes a simple yet representative motif gene circuit, and these values (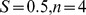,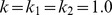) are used in many previous studies [15]–[17], [25].

#### 1. The phase of cell fate network

To explore the dynamics under different conditions mimicking by different choice of parameters, we showed the phase diagram in Figure 1(C). If we can keep the mutual repression strength 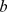 fixed and the self activation 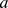 at various levels mimicking the actual developmental process where expression levels of transcription factor change [15](e.g. The expression level of transcription factor 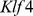 can be viewed as the effective self activation 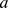 at various levels mimicking the actual developmental process [32]. Because 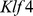 is not required for the maintenance of undifferentiated state of ES cells [32]. Furthermore, the expression level of 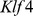 decreases gradually after induced differentiation [32].), the cells are attracted to different differentiated and undifferentiated states. There are five regions in the parameter phase space in Figure 1(C). Region I with lower self activation 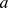 and mutual repression 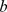 has only one stable state 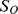 with lower equal levels of the expressions of two lineage specific genes 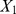 and 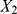 shown in Figure 1(A). This is an intermediate state phase with lower lineage specific genes in the process of transdifferentiation [4]. Region II with higher mutual repression 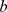 and lower self activation 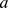 has two stable states shown in Figure 1(A): 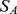 which represents the differentiated state with higher expression of 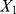 and lower expression of 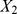, 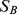 which represents another differentiated state with lower expression of 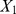 and higher expression of 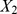. Region III with lower mutual repression 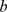 and relative higher self activation 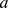 has three states: 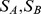 and 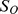. Region IV with higher mutual repression 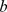 and self activation 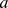 has three states: 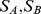 and 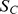 which represents a pluripotent state with medium equal expressions of 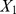 and 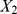 in the process of dedifferentiation which can also be viewed as the process of reprogramming. Region V with lower mutual repression 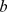 and higher self activation 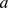 has all the four stable states: 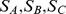 and 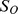.

By changing the parameters of self activation 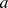 and mutual repression 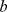, we can induce the initial differentiated cell to another differentiated cell in region II through the region III or region I by transdifferentiation (the yellow solid line), or through the region IV by dedifferentiation (the red dash line). In regions II, III, IV and V, there also exist tansdifferentiation within each. We will explore the dynamics of gene regulatory network for cell fate decision making process resulted from three driving force of stochastic fluctuations, gene regulation and induction through the instructive changes in details via the corresponding landscape topography for cell development.

#### 2. Super-critical and sub-critical pitchfork bifurcation versus saddle-node bifurcation in cell fate network

**Figure 3:**
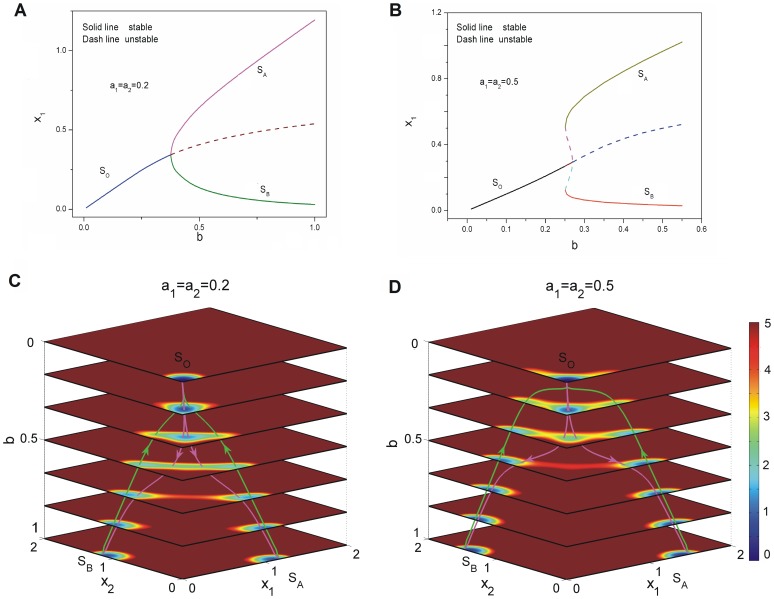
The dynamics of super-critical and sub-critical bifurcations for cell type switchings. A: The phase diagram for changing the parameter 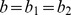 with 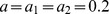. B: The phase diagram for changing the parameter 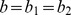 with 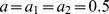. C: The quantified dedifferentiation and differentiation landscape and pathways for continuous changing parameter 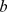 with 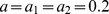. D: The quantified dedifferentiation and differentiation landscape and pathways for continuous changing parameter 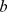 with 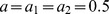.

We explored the bifurcation for cell fate decision network for different conditions. When mutual repression regulation parameters 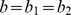 increase with small self activation regulation 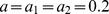, the phase diagram has a super-critical pitchfork bifurcation which is a second order phase transition [33], [34] shown in Figure 3(A). The solid lines represent stable fixed points while the dash lines represent unstable fixed points. We can see a stable state 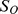 becomes an unstable state and splits into a pair of new stable states 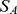 and 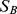 at the critical point [33], [35]. As the self activation regulation strength 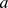 increases, the phase diagram changes to a new form of sub-critical pitchfork with two saddle-node bifurcation which is a first order phase transition shown in Figure 3(B) as 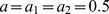. The initial state 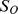 is mono stable at lower mutual repression 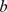, then a pair of new stable states 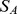 and 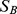 (two saddle-node bifurcations) emerge at somewhere far away from the initial state 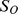 as mutual repression 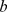 increases. After the critical point of sub-critical pitchfork, the center initial stable state 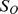 at the center becomes unstable, only the two new stable states 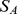 and 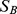 are left in the phase space. Super-critical pitchfork bifurcation represents a type of “second-order transition” in physics [36]. The difference between super-critical pitchfork bifurcation and sub-critical pitchfork bifurcation is that: super-critical pitchfork bifurcation represents one stable equilibrium splits into two stable equilibrium and a unstable equilibrium while sub-critical pitchfork bifurcation represents two unstable equilibrium and a stable equilibrium merge into an unstable equilibrium. Thus super-critical pitchfork bifurcation differs from the sub-critical one in that two new stable equilibrium 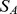 and 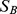, when they appear, already have a significant distance away from the middle stable equilibrium 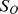. But the two stable fixed points and the two unstable fixed points in sub-critical pitchfork with two saddle-node bifurcations are both symmetric in 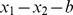 three dimensional space, while they are not symmetric in 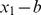 two dimensional space shown in Figure 3(B). These two bifurcations shown in Figure 3(A) and (B) are similar to the picture described in Waddington's epigenetic landscape [12].

**Figure 4:**
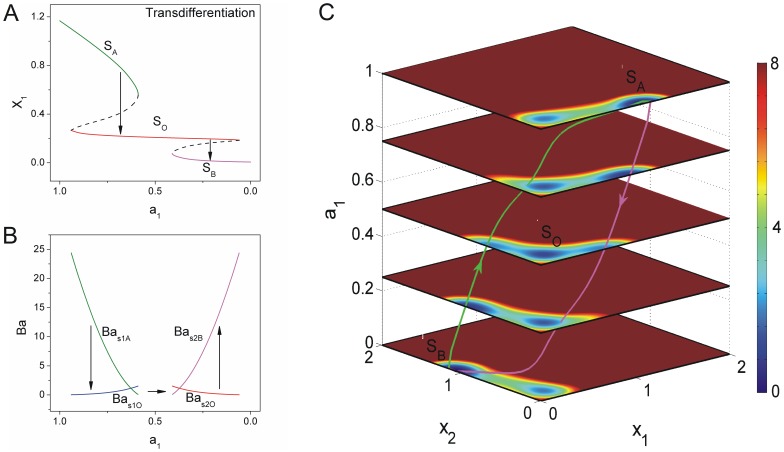
The dynamics of transdifferentiation undergoing an intermediate state. A: The phase diagram for decreasing 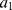 induced the differentiated state 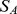 to the other differentiated state 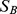 through the intermediate state 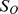. (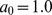, 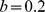) B: The barrier heights of the population landscape versus the parameter 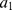. C: The quantified transdifferentiation landscape and pathways for continuous changing parameter 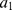.

**Figure 5:**
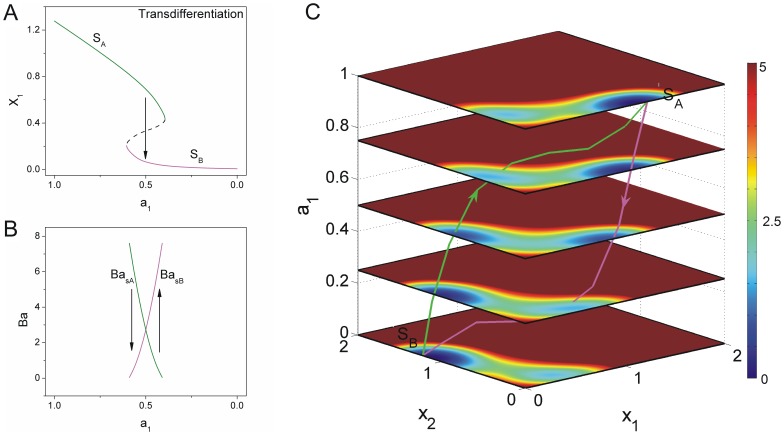
The dynamics of transdifferentiation undergoing a series of unstable states. A: The phase diagram for decreasing 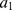 induced the differentiated state 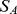 to the other differentiated state 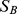. (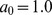, 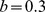) B: The barrier heights of the population landscape versus the parameter 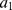. C: The quantified transdifferentiation landscape and pathways for continuous changing parameter 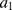.

**Figure 6:**
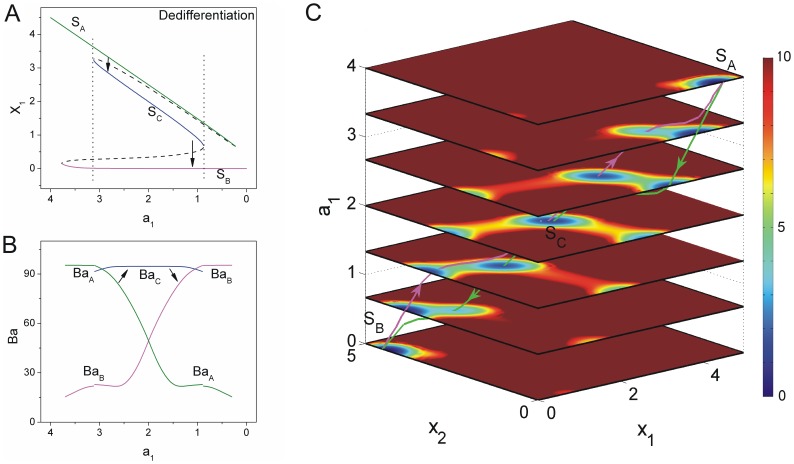
The dynamics of dedifferentiation undergoing a pluripotent state. A: The phase diagram for decreasing 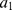 induced the differentiated state 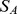 to the other differentiated state 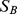 through the pluripotent state 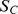. (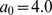, 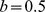) B: The barrier heights of the population landscape versus the parameter 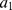. C: The quantified dedifferentiation landscape and pathways for continuous changing parameter 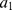.

The phase diagrams shown in Figure 4(A), Figure 5(A) and Figure 6(A) are saddle-node bifurcations. A saddle-node bifurcation denotes a collision and disappearance of two equilibria rather than a pitchfork bifurcation [33], [35]. The saddle-node bifurcation is a first order phase transition [33], [34]. We can see that the initial valley 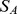 does not split into new valleys as the description of Waddingtons epigenetic landscape (a pitchfork bifurcation) [35]. New valleys 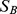 and 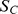 or 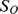 are born at somewhere far from the existing valley 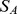 in the state space. It is anther way of creating or eliminating the valleys from the potential landscape besides a pitchfork bifurcation [35]. The cell moves to the new valley 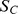 or 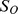 and 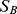 in sequence under fluctuations since its own valley disappears in another saddle-node bifurcation. We have already explored another form of bifurcation for cell fate network as self activation 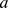 decreasing with 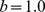 in our previous study [15], [17]. The phase diagram was drawn on the intrinsic potential landscape as the black lines in Figure 1(D) which is a sub-critical pitchfork [33], [34] at the phase transition point (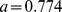).

We would like to explore these mentioned non-equilibrium phase transition under fluctuations and gene regulation. We might monitor the expressions of the differentiation marker genes in time and obtain the correlation functions. The singularity of the self-correlation function indicates the first order phase transition (saddle-node bifurcation) and the continuity of that shows the second order phase transition [33], [34]. Thus we might distinguish these mechanisms of cell type switchings. We will explore these mechanisms of four bifurcations through our potential landscape theory in details below.

#### 3. Intrinsic potential landscape

We obtained the intrinsic potential landscape 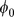 (see the section of Methods) with Lyapunov properties to quantify the global stability by solving the zero fluctuation limit Hamilton-Jacobi equation and the associated intrinsic flux velocity in the zero noise limit [37]. The population potential landscape of cell development can be obtained through the exploration of the underlying probability dynamics, by solving the Fokker-Planck diffusion equation (see the section of Methods) [15]. The population potential landscape 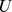 is related to steady state probability distribution 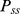 through 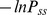 under fluctuations. The intrinsic potential landscape is quantified at the zero noise limit while the population potential landscape is quantified under finite fluctuations. Both show the global properties of the cell developmental process. Although intrinsic potential landscape gives less information (only at zero noise limit) about the network than population potential landscape, it can be used to quantify the global stability due to its nature of being a Lyapunov function [37]. We can illustrate two-dimensional potential landscape (the coordinates 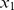 and 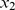) to one dimension. One dimensional cross section coordinate 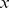 links 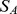 side minimum through 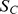 middle minimum to 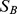 side minimum. 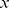 represents the gene expression levels, 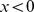 shows gene 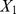 is dominant while 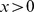 shows 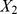 is dominant. If the self activation strength 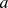 decreases relatively slowly, relative to gene regulation in development, the potential landscape can be viewed as a succession of one dimensional potential slice. Figure 1(D) shows the intrinsic potential landscape for normal cell differentiation development process from pluripotent state (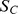) to differentiated states (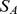 and 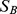) and the pluripotent reprogramming process from differentiated states (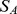 and 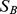) to pluripotent state (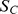). We can see the intrinsic potential landscape 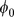 can be used to quantify the Waddington's picture and has almost the same shape with the population potential landscape [15].

The red dash lines and the yellow solid line shown in Figure 1(D) schematically described the lineage reprogramming process: dedifferentiation and transdifferentiation, respectively. The dedifferentiation process shows that differentiated state 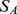 follows a step backward to a pluripotent state 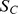 and then is induced to re-differentiate to another differentiated state 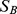. While the transdifferentiation process shows that differentiated state 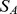 converts directly to another differentiated state 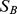 through certain intermediate stable state or not. Much work has been done on lineage reprogramming and progress has been made in manipulating the key regulator gene to convert cell lineages [3]–[6], [8], [9]. The understanding of the underlying mechanism is still challenging. We will discuss the possible mechanisms of these lineage reprogramming process in detail using this simple gene regulatory circuit.

We can see that when self activation 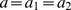 is strong with higher mutual repression 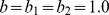, the valley of the central pluripotent state 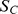 is much deeper and the system is attracted to this valley shown in Figure 1(D). As the strength of self activation 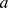 decreases, the valleys of side differentiated attractors 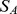 and 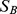 become deeper while the central pluripotent state 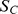 becomes weaker. When the strength of self activation 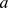 approaching to zero, the central state 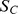 becomes a ridge and therefore it is not stable while the side states 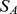 and 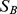 become stable. This result of intrinsic potential landscape with global Lyapunov property of global stability shows the similar mechanism with the result obtained from exploring the population potential landscape [15]–[17].

**Figure 7:**
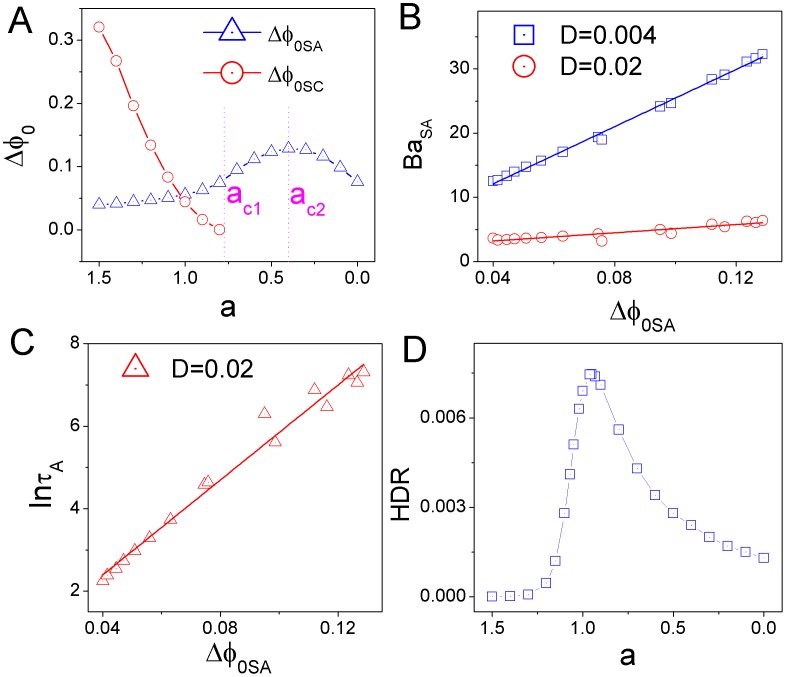
The barrier height, escape time and dissipation rate for different self activation strength 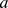 with mutual repression strength 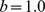 under fluctuations. A: The intrinsic barrier height 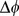 versus 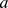. B: The intrinsic barrier height 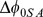 versus the population barrier height 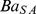 in 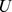 for 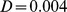 and 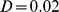. C: The escape time 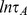 from the valley 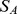 versus the intrinsic barrier height 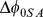. D: The dissipation rate versus the decreasing parameter 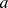.

In order to quantify the stability of each state from the potential landscape topography, we can apply barrier height to measure the relative weights between different stable states. We showed barrier height of intrinsic potential landscape versus the strength of self activation 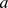 in Figure 7A. We set 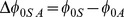 and 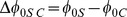, where 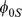 is the value of the intrinsic potential landscape at the saddle point between state 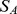 and state 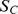, 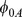 represents the minimum value of the intrinsic potential landscape at differentiated state 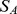 while 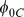 represents the value of that at pluripotent state 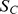. Barrier height 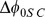 decreases as 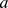 decreases, and state 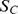 vanished after the phase transition critical point 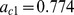, where the system transits from three stable states (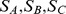) to two stable states (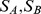). It implies that the attraction of state 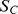 becomes shallower. Barrier height of 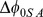 increases first, then decreases. It shows that the attraction of the differentiated state 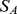 (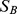) becomes deeper first, then becomes weaker after another critical point around 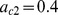. So differentiated states 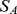 and 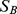 at 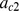 are more stable. Figure 7B shows the intrinsic potential barrier height 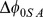 has positive correlation with the population potential barrier height 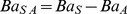 under the diffusion coefficient 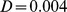 and 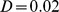, where 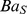 is the value of the population potential landscape at the saddle point between state 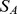 and state 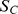, 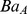 represents the minimum value of the population potential landscape at the differentiated state 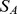. The mean first passage time (MFPT) is useful to characterize the global stability if stochastic fluctuations are the dominant source of noise since it measures how the system can globally communicate from one state to another. The intrinsic barrier height 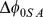 and the corresponding MFPT have the correlation of 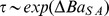 shown in Figure 7(C) with diffusion coefficient 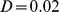.

A cell is a non-equilibrium open system with exchanges of energy and information from the outside environment. This leads to dissipation which is determined by both potential landscape and flux. The dissipation can give another global physical characterization of the non-equilibrium system. Non-equilibrium system dissipates both energy and entropy in steady state, where the entropy production rate is equal to heat dissipation rate. The heat dissipation rate is formulated as 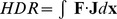 [13], [37]–[40], which increases first then decreases as self activation 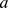 decreases as shown in Figure 7(D). This indicates that larger area of the dominant probability flux leads to more heat dissipation because the system needs to consume more energy [37]. The system consumes more energy in the process of the development with three dominant states while the system consumes less at the beginning of cell development and at the end of cell development with less states. The heat dissipation rate provides a global characterization of cell development. It is intimately related to the robustness of the underlying network.

### II. The mechanisms of cell type switchings

#### *1. Stochastic Fluctuations.* The cell type switchings at a given stage of development with different symmetric self activation 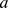 at fixed mutual repression 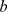


**Figure 8:**
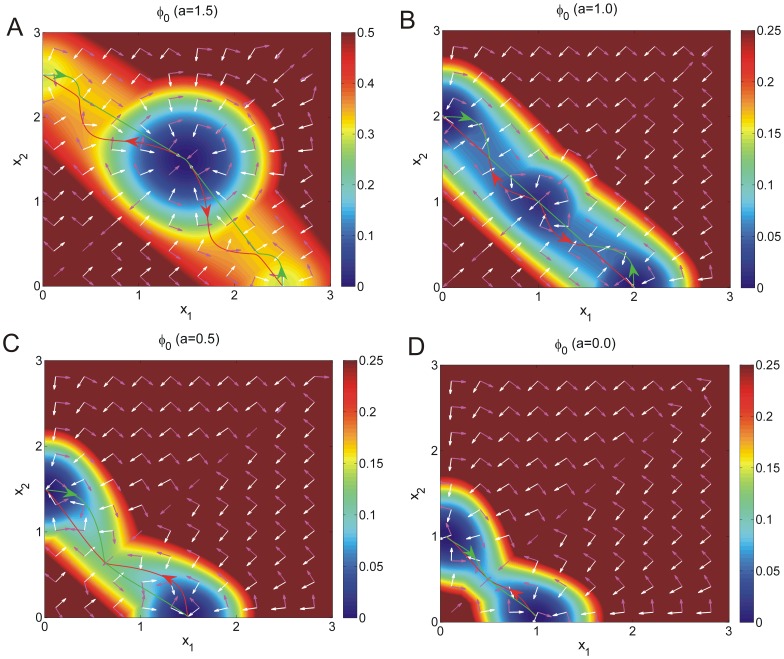
The paths of cell type switchings with different self activation strength 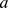. The paths of differentiation (A,B), dedifferentiation (A,B) and transdifferentiation (C,D) for different 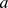 in zero-limit fluctuations on the intrinsic potential 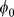. Purple arrows represent the intrinsic flux velocity (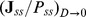) while the white arrows represent the negative gradient of intrinsic potential (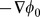)).

The stochastic or inductive cell development can often be influenced by the external environment. We showed the paths of state transitions in cell development on the intrinsic potential landscapes for different self activation 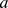 with fixed mutual repression 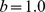 due to stochastic fluctuations shown in Figure 8. We can see the green lines represent the reprogramming or dedifferentiation paths from differentiated state 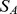 or 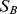 to pluripotent state 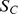 while the red lines represent the differentiation paths from pluripotent state 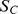 to differentiated state 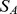 or 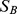 shown in Figure 8(A)(B) when self activation 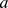 is relative stronger and the system has three stable states. Its worth pointing out that a green path from differentiated state 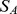 to pluripotent state 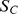 connected to a red path from pluripotent state 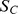 to another differentiated state 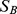 can provide a possible mechanism of the process of dedifferentiation first and then redifferentiation shown in Figure 8(A)(B). We also showed that both the green and the red lines represent the transdifferentiation paths from one differentiated state to another differentiated state shown in Figure 8(C)(D) when self activation 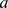 is relative weaker and the system has only two stable states, just as a toggle switch. The intrinsic flux velocity (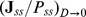) represented by purple arrows are perpendicular to the negative gradient of intrinsic potential (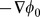) represented by the white arrows in Figure 8 (see the section of Methods).

#### The cell type switchings processes at a given stage of development with symmetric changing mutual repression 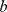 while fixing self activation 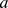


**Figure 9:**
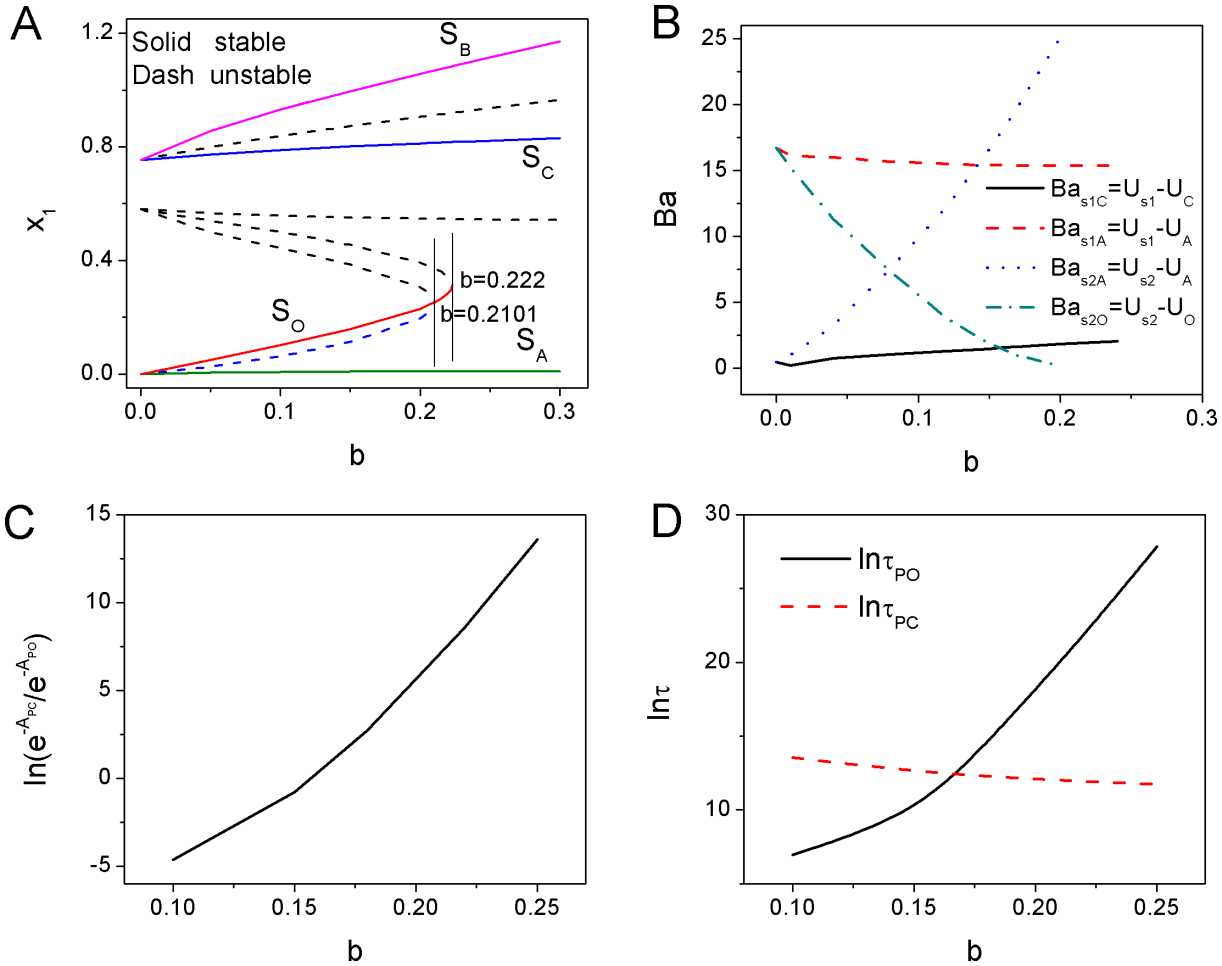
The phase diagram, barrier height, probability of the dominant path and mean first passaging time for different mutual repression strength 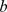. A: The phase diagram for changing mutual repression strength 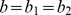 with 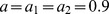. B: The barrier heights versus the parameter 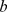. C: The probability of the dominant path through the progenitor cell state 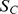 divided that of the path through the intermediate state 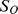 versus the inhibition strength 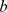. D: The mean first passaging time through the two paths versus the inhibition strength 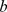.

We considered the potential landscape changing under fluctuations with varying mutual repression parameter 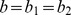 at a given state with fixed self activation 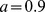. Figure 9(A) shows the phase diagram for changing mutual repression strength 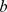. We can see that when mutual repression strength 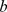 decreases below 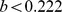, a new stable state 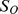 emerges. This is an intermediate stable state between differentiated states 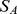 and 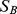. There are lower expressions of gene 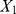 and 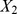 in state 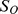. Dashed lines represent the saddle point between stable states. As mutual repression 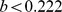, the system has all four states 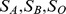 and 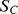. The fluctuations in the system can enable stochastic switching among the stable states. Note that smaller mutual repression strength 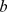 here represents larger repression effect since the parameter 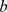 is in the numerator of an inhibition term with a positive sign. Smaller 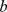, that is larger repression, leads the system towards intermediate state 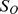, while larger 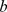 which represents smaller repression effect leads the system towards pluripotent state 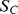.

**Figure 10:**
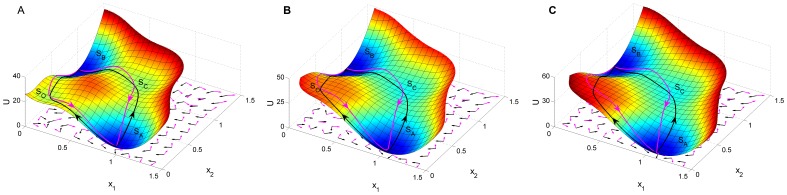
The flux on the population potential landscape. The flux on the population potential landscape with 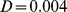. Purple arrows represent the flux (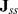) while the black arrows represent the negative gradient of population potential landscape (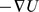)) for 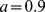, 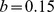 (A), 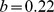 (B), 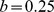 (C). The black lines represent the pathways from state 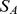 to state 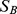 while the purple lines represent the pathways from state 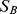 to state 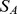.

Any given cell may take a completely different route back to their pluripotent state in principle. Certain sequence of stages can emerge in the process of cell type switchings [4]. In experiments, if there are several pathways, one can collect the statistics and find out the relative probabilities of each path, giving the quantification of the path weights. In modeling, path integral weights are calculated by the action of the system analogous to the classical mechanical systems which determine the likelihood of one path versus the other. We often used the dominant paths with the largest weights to represent the major pathways. We showed four dominant biological paths on the corresponding population landscape with different mutual repression strength 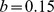 (A), 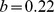 (B), 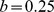 (C) in Figure 10. These processes are fluctuation or induction induced transition. The purple lines represent the paths from state 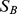 to state 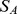 while the black lines represent the paths from state 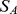 to state 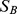 [15], [37]. We can see there are two dominant paths with the same color for transdifferentiation from a certain differentiated state to another differentiated state in each sub figures, one path is through intermediate state 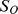 while the other path is through pluripotent state 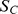. We also found the two different colored development paths between each two states follow quite different routes. It is irreversible between the forward dedifferentiation and the backward dedifferentiation paths through the pluripotent state 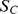, and between the two transdifferentiation paths through intermediate state 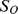 or without an explicit intermediate state. This illustrates the irreversibility of the developmental paths which can be verified from the ongoing and future dynamical experiments.

The path weight represents the probability of each route for cell type switchings. It can be used to predict the probability of different routes for cell type switchings. The path probability can be obtained by the action 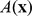 for cell development (See methods for details). We labeled 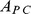 as the action of the path through state 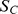, and 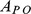 as the action of the path through state 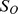. Figure 9(C) showed the logarithm of dedifferentiation path probability through state 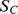 divided that of transdifferentiation through state 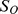 decreases as mutual repression strength 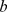 becomes weaker. This showed that the dedifferentiation path probability through state 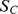 decreases or the transdifferentiation path probability through state 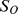 increases as mutual repression strength becomes weaker.

The purple arrows represent the direction of the probability flux 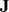 while the black arrows represent the direction of the negative gradient of population potential landscape 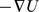 shown in Figure 10. We can see the flux is almost perpendicular to the negative gradient of the population potential landscape [13], [37]. The dynamics of transdifferentiation and dedifferentiation processes are determined by both gradient landscape and probability flux. Probability flux provides a curling force breaking the detailed balance, and leads the system to stay at the non-equilibrium state. The gradient force attracts the system into stable valleys. The potential landscape and flux both direct the processes of cell type switching. Flux can lead a system to move on even a relatively flat landscape, e.g., the limit cycle attractor, thus “flux-directed differentiation” and “down-hill-directed differentiation (Waddington)” both can occur in cell development. “down-hill-directed differentiation (Waddington)” leads to the exponential waiting of barrier crossing while “flux-directed differentiation” gives a much more precise timing. Flux also can lead the biological paths of cell type switchings to be deviated from the paths obtained by steepest descent gradient, and the corresponding paths of cell type switchings are irreversible. We would like to point out additional flux can emerge from epigenetics of slow (non-adiabatic) transcription and translation regulations [41] often encountered in eukaryotic cells. The flux generated by the slow time scales can lead to the new mechanism of differentiation and reprogramming [31], [42]. The competition of barrier crossing and slow binding can lead to optimal speed of cell type switching. [31], [42], [43].

It is worth noting that even though state 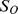 disappears in Figure 10(C), there still exist transdifferentiation paths through a series of indeterminate states near 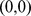 position. This provides the possible mechanism of two ways of lineage reprogramming. We labeled the saddle point between state 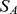 and state 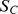 as 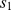 while the saddle point between state 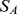 and state 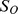 as 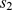. In Figure 9(B), we can see barrier height 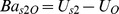 measuring the stability of intermediate state 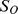 increases and barrier height 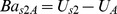 measuring the degree of difficulty for transition from state 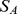 to state 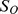 decreases dramaticlly as mutual repression strength 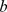 decreases, where 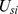 is the potential value at saddle point 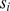 and 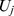 is the minimum potential value at valley 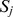. This implies that state 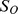 becomes more stable and robust as 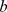 decreases.

We also can explore MFPT by 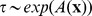 [44]. Importantly, MFPT is also useful to characterize stability of the network for changing the regulations represented by the self activation 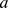 and mutual repression 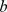 under a small but fixed fluctuations (during the regulation changes or induction) mimicking the real environments. Figure 9(D) showed MFPT along dedifferentiation and transdifferentiation paths versus mutual repression strength 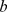. We can see that the transdifferentiation path through state 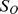 becomes more preferred than dedifferentiation path through state 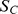, and MFPT becomes shorter for transdifferentiation path through state 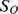 as mutual repression strength decreases. In other words, transdifferentiation process is easier (harder) and the dedifferentiation process is harder (easier) when mutual repression is weaker (stronger).

#### *2. Gene Regulation.* Decreasing self activation 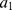 and increasing self activation 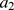 induce the transdifferentiation process from state 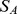 to state 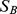 with lower mutual repression strength 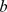


The instructive change of landscape via varying regulation strengths is another important mechanism in action for cell development. Down regulating the lineage specific gene for initial primary differentiated cell and up regulating the lineage specific gene for final target differentiated cell can induce transdifferentiation or dedifferentiation. We explored this mechanism below with changes in decreasing self activation 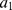 for gene 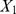 and increasing self activation 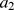 for gene 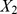.

Self activation strength can be set for describing the time evolution of the self activation regulation parameters as: 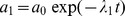 [25] which continuously decreases in time (down-regulates cell specific gene 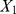 for differentiated state 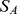) and another self activation regulation strength 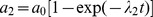 which continuously increases in time (up-regulates cell specific gene 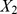 for target differentiated state 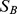) in cell developmental process due to the influences of the regulations of other genes. 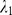 and 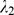 are the rates for the decrease of self activations 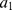 and 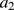. We assumed the same value of 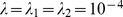 for simplicity for the latter calculations. At this value of 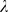, self activation strength 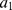 and 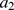 decrease relatively slowly compared with regulation dynamics of gene 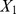 and 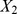. Thus the dynamics is a slow non-equilibrium relaxation process. 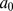 is the scaled value of self activation 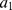 and 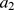 [25].

We explored the transdifferentiation mechanism below with decreasing self activation 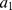 and increasing self activation 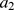 with lower mutual repression strength 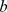. Figure 4(A) shows the saddle-node bifurcation phase diagram for decreasing self activation strength 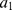 with lower 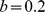 and smaller 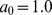. Figure 4(B) shows barrier height versus decreasing self activation 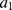 with 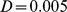. We defined the saddle point between state 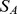 and state 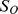 as 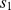, and the saddle point between state 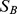 and state 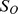 as 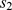. Barrier height is defined as: 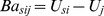, where 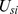 is the potential value of the 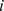 saddle point, and 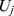 is the minimum at valley 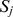. Barrier height can quantify the degree of global robust and stability at a valley. We can see the cell stays at the monostable differentiated state 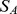 at the beginning of the transdifferentiation. As self activation 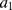 decreases, an intermediate state 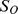 emerges. Valley 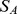 is much deeper than valley 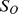 due to barrier height 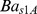 of valley 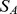 being higher than that of 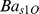. It means the differentiated state 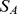 is more preferred and more attractive than intermediate state 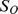. The system is preferred to stay at state 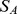 with gene 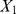 being dominant. As self activation strength 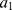 becomes weaker and self activation 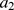 becomes stronger, the valley of state 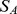 becomes shallower while the valley of state 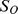 becomes deeper. Then, the valley of state 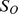 is more attractive than that of state 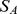 since barrier height 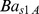 is lower than barrier height 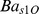, and gene 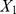 and 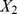 are both at lower expressions. After state 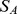 disappears, the cell is driven into intermediate state 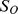. As self activation strength 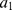 decreases further, the other differentiated state 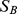 emerges, and barrier height 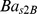 becomes higher than barrier height 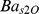. Finally, the cell is forced into state 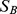. This process interprets the mechanism of transdifferentiation from state 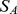 to state 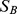 through an intermediate state 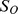.

The above results showed the dynamics at certain stage of transdifferentiation. We can also explore the continuous dynamics controlled by the set of equations below:
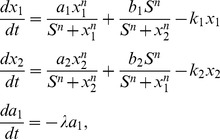
(2)where 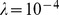 and 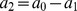. The continuous time dynamics of down-regulating gene 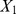 and up-regulating gene 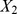 is shown in Figure 4(C) with 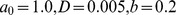 using Eq.2. We obtained the transdifferentiation paths on the four dimensional potential landscape. The purple path is from state 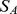 to state 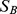 while the green path is the reverse transition both through intermediate state 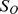. It implies that the system with small mutual repression strength 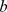 (large inhibition) prefers the transdifferentiation path through intermediate state 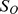. Although transdifferentiation process does not seem to occur naturally, it has been observed in many experiments. For example, the exocrine cells in adult mice can transdifferentiate into 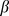-cells using defined factors for direct reprogramming without passing through a pluripotent state but through an unnatural intermediate state [4], [8], [9].

Figure 5(A) shows the phase diagram of saddle-node bifurcation under 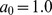 and mutual repression 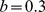. Figure 5(B) shows barrier height versus self activation 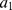 with 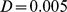. We defined barrier height as 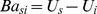, where 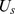 is the potential value of saddle point 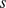 between state 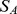 and state 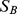, and 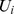 is the potential value at state 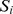. Figure 5(C) shows the paths and the landscape for continuous dynamics using Eq.2 with 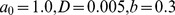. We can see the cell stays at differentiated state 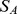 with higher barrier height 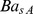 at first, then the landscape valley tilts the cell from state 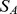 to state 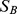, barrier height 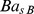 becomes higher than barrier 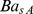 and the valley of state 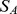 eventually disappears. Finally, valley 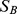 becomes deeper. The weights of these two valleys exchange at the end of transdifferentiation process [35]. This process interprets the mechanism of transdifferentiation from state 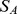 to state 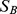 directly without through a specific intermediate state but through a series of indeterminate states. This result can be used to explain the mechanism that the enforced expressions of 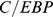 with endogenous 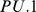 can reprogram B cell into macrophages [4], [5]. B cell specific marker is 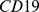 while the macrophage specific genes is 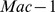. The gene regulatory circuit is shown in Figure 2(B). B cell commitment factor 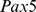 can up-regulate many B cell specific genes (such as 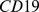). The macrophage commitment factor 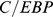 can up-regulate many macrophage cell specific genes (such as 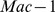) and down-regulate B cell specific genes (such as 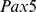) [4], [5]. Transcription factor 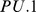 is needed in the process of transdifferentiation. The gene 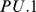 has the property of auto-activation. Mikkola's work indicated that 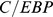 and 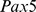 act in mutual antagonisms [5], [45]. The dashed lines for the auto-activation indicate uncertainty in Figure 2(B). Thus we can reduce the gene regulatory circuit in to two markers of 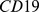 and 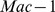 similar as our mutual antagonistic and self activation 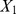 and 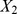 [4], [5]. 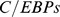 inhibit B cell commitment transcription factor (B cell-specific genes) which down-regulates B cell marker 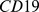 (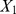) in B cell, and co-activate macrophage specific genes which up-regulates its target marker 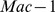 (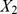) in macrophages. B cells pass through a series of indeterminate states with lower expressions of B cell-specific genes 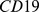 (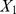) and macrophage-specific genes 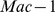 (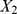), which does not seem to undergo an initial dedifferentiation [4], [5].

**Figure 11:**
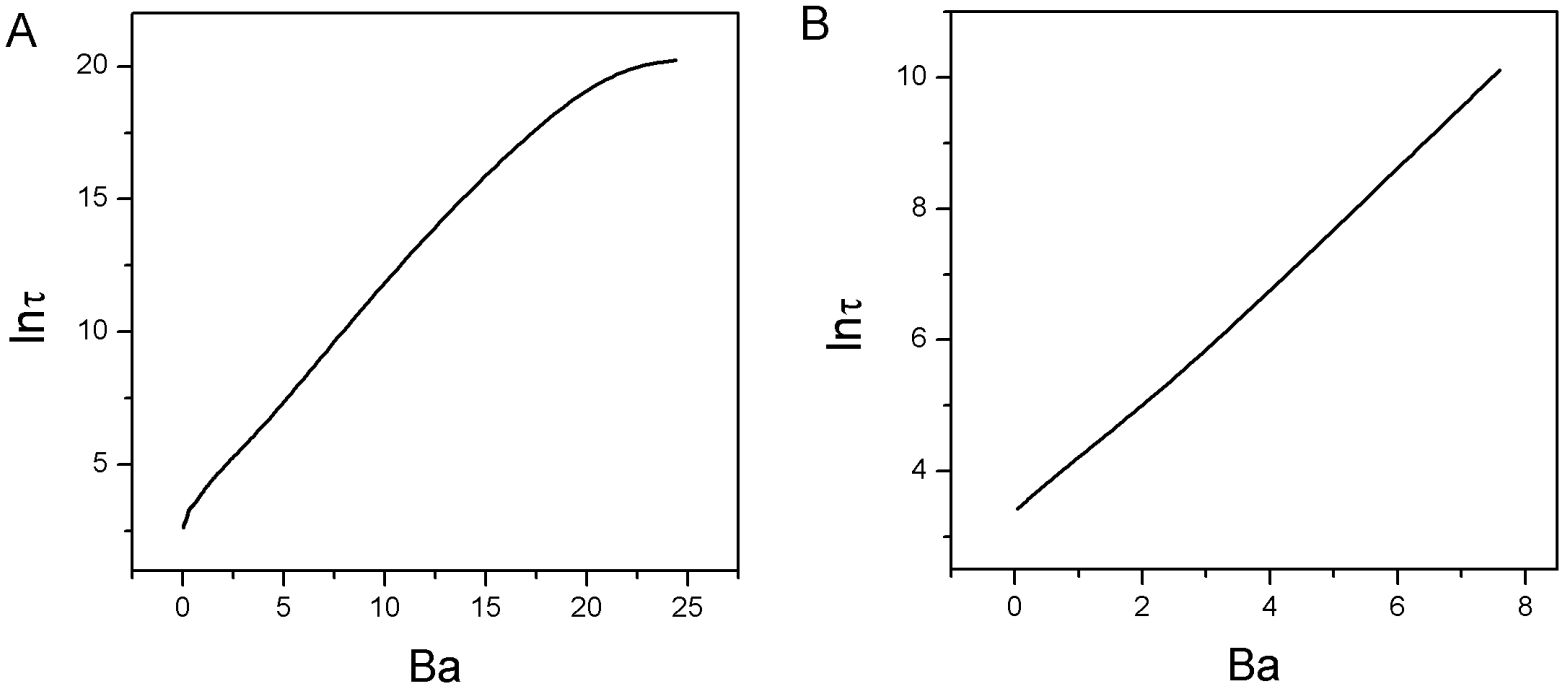
Mean first passage time versus barrier height with different mutual repression strength 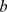. A: The logarithm of the mean first passage time (MFPT) versus the barrier heights according to Figure 4(B). B: The logarithm of the mean first passage time (MFPT) versus the barrier heights according to Figure 5(B).

Figure 11(A)(B) show the logarithms of MFPT versus barrier heights using the same parameters in Figure 4(B) and Figure 5(B) respectively. We can see the time spent from one state to another and barrier height have the relationship as: 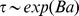. It implies that the harder the system is out from one valley with higher barrier height, the longer the escape time is.

We also explored the behavior for the system when regulation is not symmetrical, not only for the case when self-activation strength 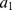 is not equal to self-activation strength 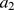, but also for the case when self-activation strength 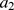 is not changing synchronously with self-activation strength 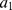. In Figure S2 in File S1, we showed the potential landscape of continuous dynamics with self-activation strength 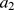 set as a constant (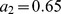) while the self-activation strength 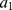 continuously decreases. The other parameters are diffusion coefficient 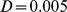, mutual inhibition strength 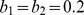. We can see the cell may stay at differentiated state 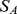 at first since the basin of differentiated state 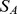 is lower than differentiated state 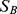 when self-activation strength 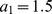, then the landscape basin tilts the cell from the differentiated state 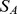 to the intermediate state 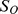, and the basin of state 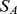 eventually disappears. Finally, the basin 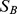 becomes deeper, and the system shifts from the intermediate state 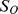 to the differentiated state 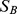. The green path is from state 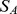 to state 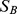 while the purple path is the reverse transition from state 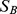 to state 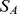 both through the intermediate state 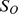. In Figure S3 in File S1, we showed the potential landscape of continuous dynamics with self-activation strength 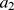 set as a constant (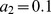) while the self-activation strength 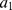 continuously decreases. The other parameters are diffusion coefficient 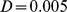, mutual inhibition strength 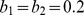. We can see the cell may stay at differentiated state 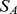 at first when self-activation strength 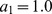, then the cell shifts from the differentiated state 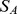 to the intermediate state 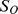, and eventually the basin of state 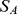 disappears. The green path is from state 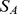 to state 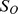 while the purple path is the reverse transition from state 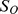 to state 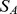. Here, intermediate state 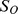 may represent the partially reprogrammed cells.

#### Decreasing self activation 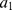 and increasing self activation 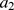 induce dedifferentiation process from state 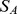 to state 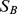 with higher mutual repression strength 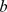


We assumed self activation 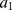 and 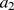 at relatively higher average scaled values with 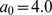 and relative larger mutual repression strength 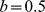 to induce the initial cell undergoing through a balanced pluripotent state [24]. Figure 6(A) showed the saddle-node bifurcation phase diagram for decreasing self activation strength 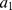 with 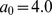 at different time. Figure 6(B) showed the barrier height versus the parameter 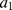 with 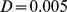. We defined the barrier height as 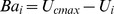, where 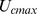 is a constant relative maximum value of population potential landscape and 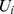 is the minimum value of population potential at valley 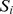. We can see the system begins with a monostable differentiated state 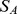 with higher expression of cell-specific gene 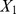 and lower expression of cell-specific gene 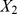. As parameter 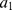 decreases, a saddle node bifurcation emerges, giving rise to another differentiated state 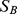 with lower expression of cell-specific gene 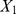 and higher expression of cell-specific gene 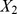. Initially, barrier height 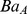 of valley 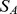 is much higher than that of valley 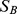, thus valley 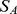 is much more stable than valley 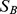. So the system prefers to stay at differentiated state 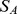. As 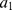 becomes weaker and the corresponding 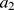 becomes stronger, two self activations 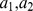 for two cell specific mutually exclusive genes 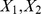 are over-expressing balanced (relative higher expression), another stable pluripotent state 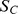 with medium expressions of gene 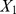 and 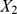 emerges, and the potential landscape has three valleys. Valley 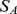 quantified by barrier height 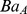 is deeper than valley 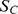 and valley 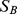 quantified by barrier height 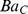 and 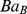 at the beginning of valley 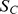 emerging. As self activation 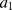 decreases and 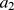 increases further, valley 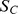 and valley 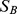 become deeper while valley 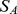 becomes shallower. Barrier height 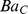 is higher than 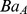 and 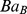 at 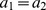. Therefore, the system with differentiated state 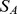 shifts to under pluripotent state 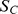 as a process of dedifferentiation. A recent experimental studies [24] proposed a model for the coupled pluripotency module (self-activation of 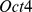 and 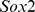) and for the differentiation module with mutual antagonism between the 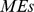 (mesendodermal) and 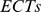 (ectodermal) shown in Figure 2(C). 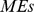 inhibit the activation between 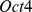 and 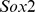, then 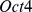 can only activates gene 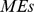, and inhibits gene 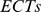 [24]. This process can be viewed as 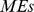 have the effect of self activation. Thus, this module can be reduced to two mutual antagonism gene 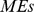 and 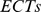 with indirect self activation as our gene regulatory circuit of 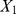 and 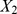 shown in Figure 2(C). It implies that higher self activation strength 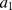 and 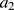 being balanced can lead the differentiated cell back towards the pluripotent cell. As self activation 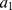 keeps on decreasing and 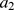 keeps on increasing, the valley of the other differentiated cell state 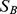 becomes deeper than that of pluripotent cell state due to barrier height 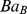 being higher than 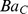 and 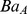. Eventually, the valleys of 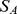 and 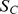 disappear at their saddle-node bifurcation [35]. Thus the cell leaves the pluripotent cell state 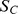 and is forced to enter into the other differentiated cell state 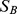. The results showed the mechanism of dedifferentiation and redifferentiation. This mechanism can be used to explain many studies of cell dedifferentiation process during tissue regeneration both in vitro and in vivo [6]. For example, 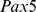 is essential for initiating B cell commitment and is continuously required to maintain B cell lineage commitment [6], [7], [45]. 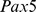 deletion can convert committed B cells into hematopoietic progenitors with pluripotency [6], [7], [45]. It is partly similar as the circuit in Figure 2(B) if we substitute 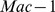 into other lineage specific genes. 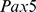 deletion means down-regulating the B cell specific genes (such as BCs) as the effect of self activation 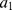. This gene regulation can lead B cells (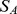) to dedifferentiate to hematopoietic progenitors (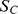). Then these cells can re-differentiate to T cell, macrophage or granulocyte (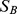) under appropriate culture conditions, such as the T-cell-deficient circumstance to reconstitute T cell development [6], [7]. The appropriate culture conditions can be achieved by up-regulating the target cell genes as the effect of another self activation 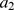.

The population potential landscape at different developmental stage of decreasing self activation parameter 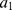 after the relaxation process to a steady state among 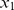 and 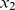 is shown in Figure 6(C) using Eq.2. The green line represents the dedifferentiated path from differentiated state 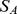 to another differentiated state 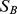 through pluripotent state 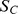. The purple line represents the backwards dedifferentiated path from differentiated state 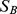 to another differentiated state 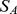 also through pluripotent state 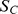. We can see the irreversible paths on the four dimensional population potential landscape due to non-zero flux. The dedifferentiated landscape and the paths can be quantitatively described for predictions.

#### Decreasing mutual repression strength 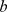 induces differentiation and dedifferentiation process from state 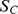 to state 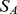
(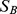) with certain self activation 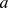


Figure 3(A) shows the phase diagram of super-critical pitchfork bifurcation under self activation 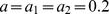 while changing mutual repression strength 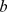. We can see the potential landscape of continuous dynamics shown in Figure 3(C) using Eq.2 with 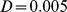, self activation 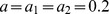 and decreasing mutual repression 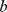 is similar to Waddington's epigenetic landscape [12], [35]. A cell valley can form from an undifferentiation state around 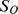. 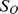 can be viewed as a stem cell state with lower expressions of differentiation gene markers while 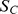 can be viewed as the stem cell with medium expressions of the stem cell markers [17], [46]. When decreasing mutual repression strength 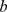, the initial valley splits into two other valleys and the initial valley becomes a ridge [35]. The cell will choose one valley as its fate. Figure 3(B) also shows the phase diagram of another form of sub-critical pitchfork with two saddle-node bifurcation under larger self activation strength 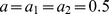 when increasing mutual repression 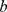. The continuous potential landscape shown in Figure 3(D) using Eq.2 with 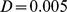, self activation 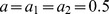 and decreasing mutual repression 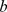 is also similar to Waddington's epigenetic landscape [12], [35] except the surrounding of the critical point. Around the critical point, there coexist three stable states 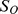, 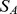 and 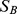. We also quantified the paths on the potential landscapes. The purple lines represent the differentiation paths from undifferentiation state to differentiation state while the green lines represent the dedifferentiation or reprogramming paths. We can see the paths are irreversible even in the pitchfork bifurcation due to the existence of flux. This mechanism can describe the autonomous cell fate specification [47]. Stem cells must fulfill two tasks of self-renewal and generation of differentiated cells. In symmetric cell division, each stem cell can divide to generate either two daughter stem cells or two differentiated cells symmetrically while in asymmetric cell division, each stem cell splits to one daughter stem cell and one daughter differentiated cell [48]. The pitchfork bifurcation in this study can represent an asymmetry event that a polarized mother stem cell splits into two daughter cell 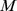 or 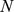 with different expressions of 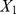 or 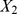. If daughter cell 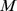 with a very low value of 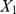 or 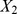, it might fall into differentiated state, while daughter cell 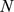 with relative higher expression of 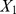 or 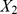 still stays at the pluripotent state [35], [48]. The asymmetric cell division usually occurs early in embryogenesis [35], [47].

#### *3. Induction of over expression*

Cell fate is influenced by inductive stimulus from a group of surrounding cells [23], [35]. Over-expressions of defined transcription factors can induce one cell type to another cell type which does not depend on gene regulations. This has been achieved in practice using over expression of stem cell marker transcription factors. In our previous gene circuit studies of cell fate decision making for stem cell differentiation and development [15], [17], the two genes in the network are both differentiation markers. The idea is that the specific differentiation markers when imbalanced will give differentiation of one cell fate or the other (two side basins 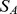 and 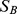). A more balanced differentiation marker setup (between the two) will lead to iPS stem cell state (center basin 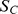). Our theoretical work [15], [17] has predicted the possibility of the seasaw mechanism (balance or imbalance) of reprogramming. That is over-expressing both the concentrations of differentiation marker genes in a balanced way can induce and force differentiated cells into iPS stem cells or pluripotent cells [15], [17].

The two mutually exclusive differentiation markers 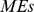 (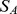) and 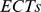 (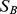) shown in Figure 2(C) with balanced over-expressions of key transcription linage specific factors can induce the lineage cell into pluripotent state (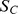) instead of the stem cell markers 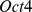 and 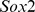 for pluripotency of reprogramming as a “seesaw model” [24]. Our theoretical work [15], [17] has already predicted this possibility of expressing differentiation markers for reprogramming and the seasaw mechanism suggested in their work [24].

Significant efforts have been made towards the experimental converting fibroblasts (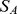) to cardiomyocytes (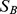) by induction of over-expressing key genes. It is reported that direct transdifferentiation can be achieved by over-expressing gene 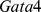, 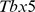 and 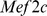 from fibroblasts (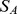) to cardiomyocytes (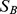) [11]. 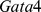, 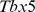 and 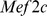 are core transcription factors during early heart development and can co-activate other cardiac gene expression [11]. So 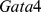, 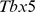 and 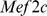 can be viewed as cardiomyocyte cell specific gene 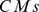 which have self activation. Over-expressing gene 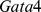, 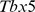 and 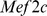 (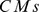) can transdifferentiate fibroblasts to cardiomyocytes not through a pluripotent state [11]. Another experiment showed that the indirect transdifferentiation can be achieved with an initial dedifferentiation from fibroblasts (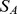) through pluripotent precursor-Cardiac progenitor (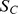) by over-expressing some stem cell markers 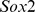,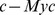,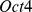 and 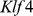, and then be induced to cardiomyocytes (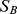) [10].

## Conclusions

In this study, we applied our potential and flux framework to explore the mechanisms of cell developmental processes of differentiation, dedifferentiation, reprogramming and transdifferentiation. The potential landscape of two gene regulatory circuit shows that the system has four stable valleys at specific regulation regions, two differentiated state 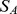 and 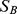, one pluripotent state 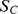, and an intermediate state 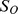. Our work provides a quantitative basis for explaining the mechanisms of the transition among the four states. Barrier height based on the population potential landscape or the intrinsic potential landscape can quantify the stability of the attractors and the efficiency of switching among the attractors. We can acquire the dynamical transition rate of the system from one valley of attraction to another by MFPT for escape and the dominant paths for dedifferentiation and transdifferentiation via the path integral method. We can see the paths of cell type switchings are irreversible due to non-zero probability flux.

In this study, we have discussed three driving forces: stochastic fluctuations, gene regulation and induction, which can lead to cell type switchings. The cell type switching driven by stochastic fluctuations is a spontaneous transition, gene regulation is much like a non-autonomous varying of time-dependent landscape, and induction is a condition of initial value re-setting process with no apparent paths. The fluctuations maybe small in some cases but never zero. When exploring the stochasticity, we used fixed set of the values of self activation and mutual repression regulation parameters 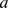 and 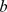. We not only discussed the possibility of cell type switching through stochastic dynamics but also other two mechanisms including the induction and regulation changes. We also explored the different dynamics with different sets of the parameter 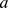 and 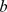. For gene regulation, we varied the parameters 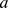 and 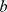 for regulating the cell type switchings. For induction, we did not change any parameters. Instead, we just gave the cell an initial set (condition) with over-expression of its lineage specific gene. We quantitatively investigated the mechanism of cell type switching through the induction without the change of the underlying landscape and through the changes in regulations leading to the changes of the underlying landscape topography. Furthermore, these two types of cell type switchings driven by gene regulation and induction are not spontaneous transitions only due to fluctuations, but a controlled process under either the changes in regulations with regulation-dependent potential landscape or the induction with fixed potential landscape.

We found that the topography of the global potential landscapes is strongly correlated to the self activation strength and the mutual repression strength of the transcription factors. Dedifferentiation can be induced by the core regulators of pluripotent genes using in iPS or the synergistic effect of lineage specifiers in specification of differentiated cells. We can adjust two self activation strength 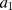 and 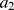 to be relative larger to force the differentiated cell to a pluripotent cell with higher inhibition strength 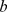, and then re-differentiate the pluripotent cell to our target differentiated cell type [24]. This process can be viewed as an initial epigenetic activation phase representing the redifferentiation after a temporal overexpression of pluripotent reprogramming factors to a pluripotent state [4], [24], [49]. Somatic cells can be transdifferentiated by temporal over-expressions of pluripotent reprogramming transcription factors. Transdifferentiation can be induced by down regulating the lineage specific marker gene (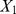) of the original differentiated cell (decreasing self activation 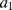) while activating another lineage specific marker gene (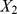) of the final differentiated cell (increasing self activation 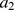) at relative lower inhibition strength 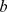, through an intermediate state or a series of indeterminate states. This process can be viewed as lineage-instructive transcription representing the induction of lineage specific gene for the target differentiated cells [4], [49]. This gives us a new understanding that the topography of underlying potential landscapes in cell development dynamics determines the feasibility and efficiency of cell type switchings.

We also classified the mechanisms of pitchfork bifurcations depicted Waddington's epigenetic development landscape including super-critical pitchfork bifurcation, sub-critical pitchfork bifurcation, sub-critical pitchfork with two saddle-node bifurcation, and saddle-node bifurcation depicted the transdifferentiation landscape [35]. We uncovered a pitchfork bifurcation of Waddington's epigenetic landscape and the irreversible paths (caused by the non-equilibrium flux) between differentiation and reprogramming. We also uncovered the saddle-node bifurcation landscape. Saddle-node bifurcation can give the explanation of possible mechanisms of dedifferentiation and transdifferentiation processes and can further explain the irreversibility of the paths for differentiation, dedifferentiation, reprogramming and transdifferentiation processes as hysteresis loop even without the presence of the non-equilibrium flux. We noticed a special kind of sub-critical pitchfork with two saddle-node bifurcations also shares the certain features with saddle-node bifurcation (hysteresis loop) and certain features of pitchfork bifurcation (Waddington's landscape).

Importantly, we uncovered some novel mechanisms as a starting point to decipher the mysterious code of the cell type switchings. Our theory can be used to guide the designs of the differentiation, dedifferentiation, reprogramming and transdifferentiation processes.

## Methods

### Quantifying non-equilibrium potential landscape, flux, non-equilibrium thermodynamics and the paths

Fluctuations exist widely in biological systems [13], [50]–[55]. The dynamics in noisy fluctuating environments can be formulated as: 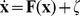. 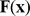 is the deterministic force, where 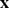 is the vector representing different concentrations in state space. 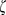 is Gaussian noise term and its autocorrelation function is 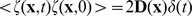, where 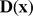 is diffusion coefficient matrix. We set 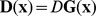, where 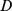 is the diffusion coefficient representing the level of noise strength while 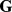 is the scaled diffusion matrix described the anisotropy phenomenon. We can explore the corresponding Fokker-Planck diffusion equation [56], [57] for probability distribution 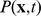: 
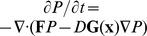
. In this study, we set 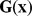 as a unit matrix for simplicity. The probability flux 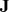 is defined as: 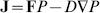. In steady state, the force decomposition is shown as: 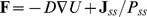 [13], [50], [51].

We obtained the Lyapunov function 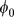 as the intrinsic potential from the zero fluctuation limit Hamilton-Jacobi equation(HJE) [37], [58]: 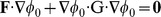 by a numerical method - level set method using the Mitchell's level-set toolbox [59]. The force decomposition in zero fluctuation limit is shown as: 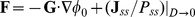. From the Hamilton-Jacobian equation above, we can obtain 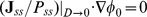 [37], [51], [60], [61]. We also can obtain the mean first passage time from the following equation [56]: 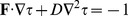. 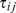 represents the mean first passage time from state 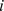 to state 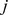.

The path integral approach we used is shown as below. The path probability starts from initial state 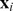 at 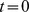, and end at the final state of 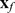 at time 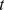. The path integral formula is shown as [15], [44] 
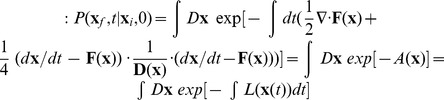
, where 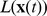 is the Lagrangian and 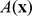 is the action for each path [15], [44]. The path integral over 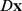 represents the sum over all possible paths connecting 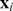 at time 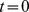 to 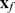 at time 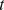. The exponent factor gives the weight of each specific trajectory path. The probability from initial state to the final state is equal to the sum of all possible paths with different weights. Every dynamical path doesn't contribute to the same weight and each path is exponentially weighted. Therefore, the path integrals can be approximated with a set of dominant paths while the other subleading path weights can be neglected for their relative small values. We can find the dominant paths with the optimal weights through minimization of the action 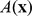 or Lagrangian 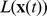. Thus, we can identify the optimal paths which give more contribution to the weight as biological paths or cell type switching pathways in our study.

## Supporting Information

File S1
**Supporting figures. Figure S1,** A: The phase diagram for varying parameter 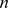 with 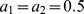, 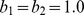, 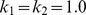 and 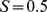. B: The phase diagram for varying parameter 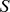 with 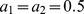, 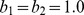, 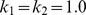 and 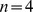. **Figure S2,** The quantified transdifferentiation landscape and pathways for continuous changing parameter 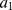 and constant 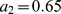. (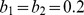, 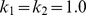, 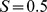 and 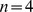). **Figure S3,** The quantified transdifferentiation landscape and pathways for continuous changing parameter 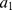 and constant 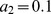.(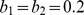, 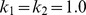, 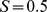 and 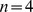).(DOC)
