# Supplementary material for: Exploring the Mechanisms of Differentiation, Dedifferentiation, Reprogramming and Transdifferentiation
Source: PLoS One. 2014 Aug 18;9(8):e105216. doi: 10.1371/journal.pone.0105216 (PMC4136825; doi:10.1371/journal.pone.0105216)

**Supporting Information**

**Figure S1.** A: The phase diagram for varying parameter n with a_1_ = a_2_ = 0.5, b_1_ = b_2_ = 1.0, k_1_ = k_2_ = 1.0 and S = 0.5. B: The phase diagram for varying parameter S with a_1_ = a_2_ = 0.5, b_1_ = b_2_ = 1.0, k_1_ = k_2_ = 1.0 and n=4.
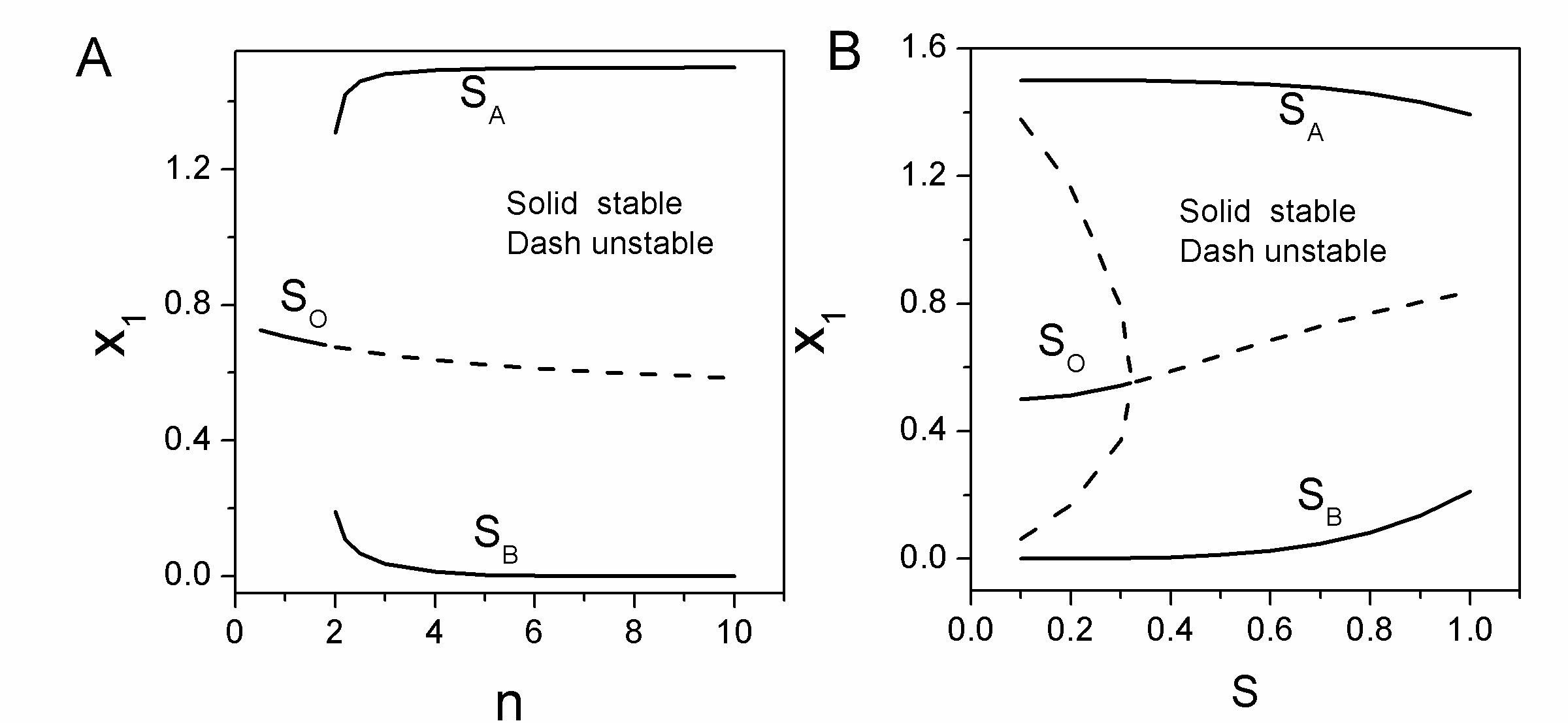


**Figure S2.** The quantified transdifferentiation landscape and pathways for continuous changing parameter a_1_ and constant a_2_ = 0.65. ( b_1_ = b_2_ = 0.2, k_1_ = k_2_ = 1.0, S=0.5 and n=4)


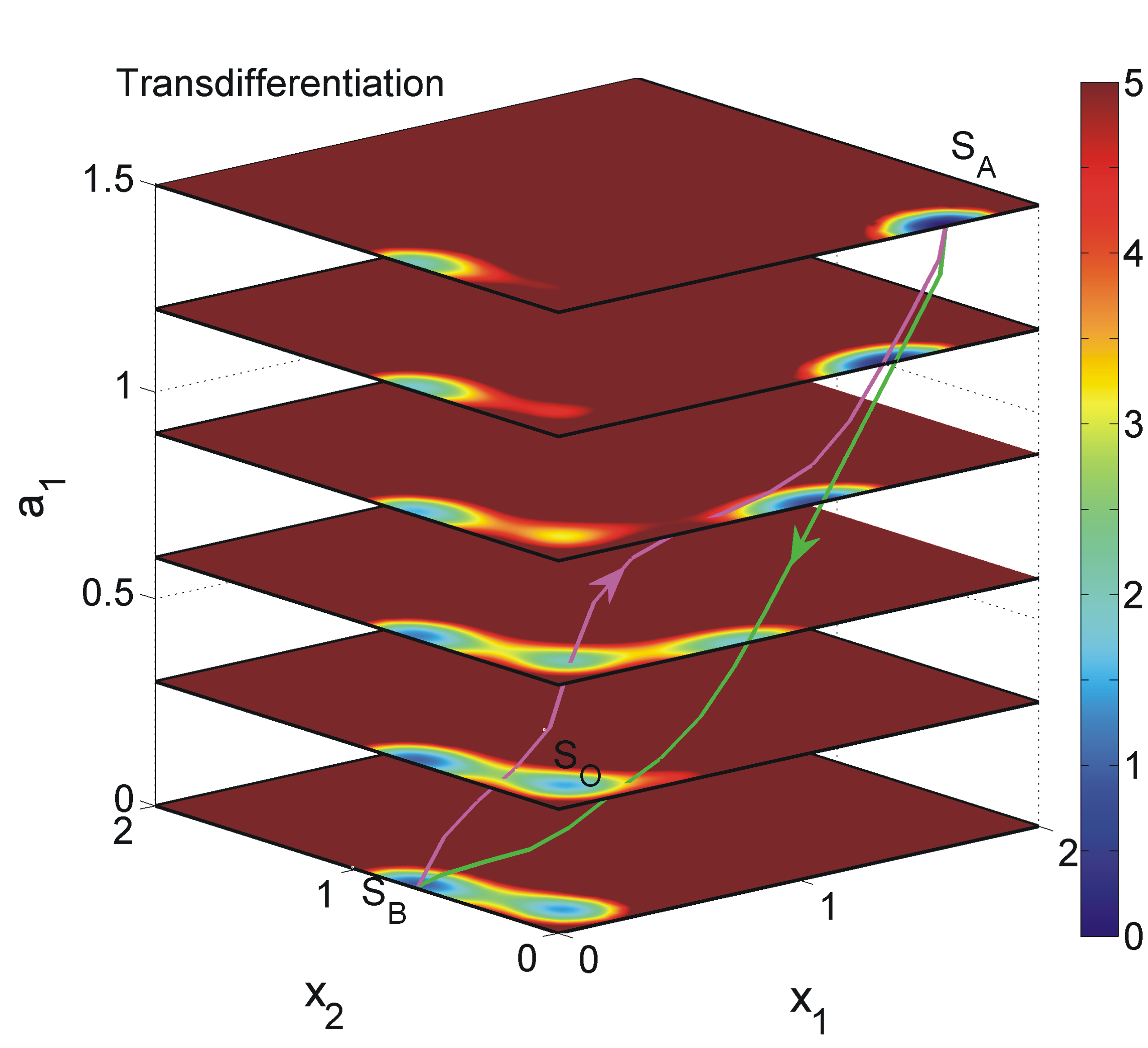


**Figure S3.** The quantified transdifferentiation landscape and pathways for continuous changing parameter a_1_ and constant a_2_=0.1. ( b_1_ = b_2_ = 0.2, k_1_ = k_2_ = 1.0, S=0.5 and n=4)


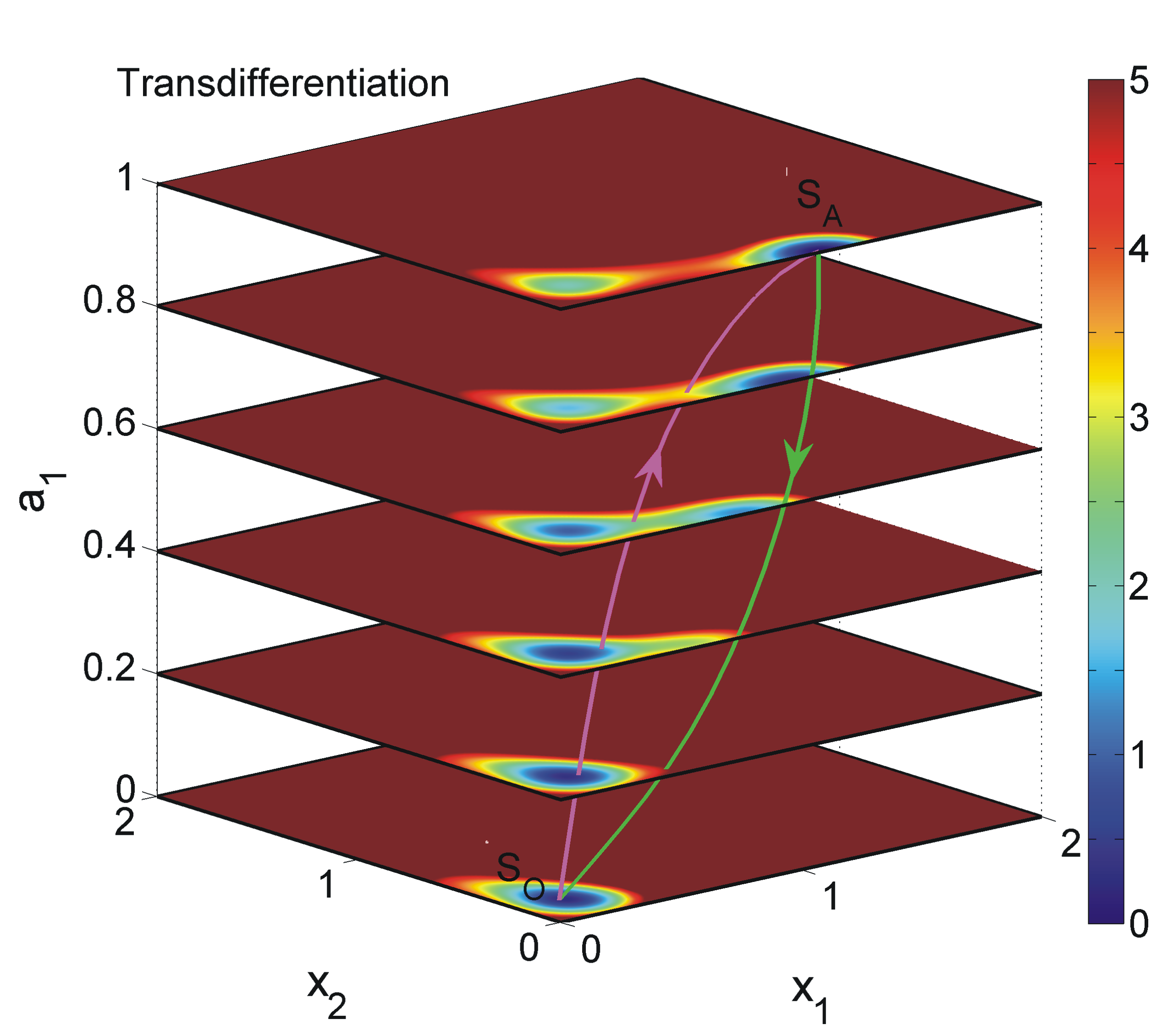

Supplement: File S1 — Supporting figures. Figure S1, A: The phase diagram for varying parameter with , , and . B: The phase diagram for varying parameter with , , and . Figure S2, The quantified transdifferentiation landscape and pathways for continuous changing parameter and constant . (, , and ). Figure S3, The quantified transdifferentiation landscape and pathways for continuous changing parameter and constant .(, , and ). (DOC) [file pone.0105216.s001.doc]
